# Supplementary material for: Molecular, Chemical, and Sensory Attributes Fingerprinting of Self-Induced Anaerobic Fermented Coffees from Different Altitudes and Processing Methods
Source: Foods. 2022 Dec 7;11(24):3945. doi: 10.3390/foods11243945 (PMC9777685; doi:10.3390/foods11243945)
Supplement: Supplementary file 1 [file foods-11-03945-s001.zip › foods-1967344-supplementary.pdf]

Table S1. Relative concentration of volatile compounds detected at T0, TF, and roasted samples of all altitudes.

Groups	*Compound*	***Code***	*%800 N T0*	*%1200 N T0*	*%1400 N T0*	*TF*			*R*			*%800 P N T0*	*%1200 PN T0*	*%1400 P N T0*	*TF*			*R*		
Acids	Acetic acid	1Ac	4.00	1.68	0.99	8.40	9.93	11.57	13.36	18.34	18.65	12.39	5.01	1.96	7.08	4.58	4.97	14.27	17.79	18.56
Acids	Heptanoic acid	2Ac	0.00	0.00	0.00	0.16	0.10	0.10	0.00	0.00	0.00	0.00	0.00	0.00	0.78	0.75	0.60	0.00	0.00	0.00
Alcohols	(S)-3-Ethyl-4-methylpentanol	2Al	8.68	8.29	8.78	2.69	3.48	2.86	0.00	0.00	0.00	1.99	2.37	2.56	0.00	0.00	0.00	0.00	0.00	0.00
Alcohols	1,6-Octadien-3-ol, 3,7-dimethyl-	3Al	3.89	2.73	2.09	2.85	1.37	0.86	0.00	0.00	0.00	3.68	3.17	2.60	0.79	0.56	0.34	0.00	0.00	0.00
Alcohols	1-Dodecanol	4Al	0.00	0.00	0.00	0.04	0.03	0.04	0.00	0.00	0.00	0.00	0.00	0.00	0.12	0.10	0.00	0.00	0.00	0.00
Alcohols	1-Heptanol	5Al	0.00	0.00	0.00	0.12	0.13	0.07	0.00	0.00	0.00	0.00	0.00	0.00	1.56	0.84	2.21	0.00	0.00	0.00
Alcohols	1-Hexadecanol	6Al	0.00	0.00	0.00	0.04	0.07	0.08	0.00	0.00	0.00	0.00	0.00	0.00	0.18	0.13	0.00	0.00	0.00	0.00
Alcohols	1-Hexanol	7Al	3.78	3.65	4.16	1.60	2.83	1.70	0.00	0.00	0.00	5.81	6.28	6.77	14.55	15.54	12.18	0.00	0.00	0.00
Alcohols	1-Hexanol, 2-ethyl-	8Al	3.78	7.33	2.03	0.00	0.00	0.00	0.00	0.00	0.00	2.88	6.62	5.77	13.49	0.00	0.00	0.00	0.00	0.00
Alcohols	1-Octanol	9Al	0.17	0.11	0.07	0.40	0.23	0.17	0.00	0.00	0.00	0.28	0.27	0.23	1.03	1.29	0.93	0.00	0.00	0.00
Alcohols	1-Octen-3-ol	10Al	5.23	5.95	6.42	0.00	2.56	3.00	0.00	0.00	0.00	1.61	3.88	4.71	0.00	11.24	8.55	0.00	0.00	0.00
Alcohols	1-Pentanol, 3,4-dimethyl-	11Al	0.00	0.50	0.51	0.25	0.46	0.23	0.00	0.00	0.00	0.14	0.12	0.26	0.00	0.00	0.00	0.00	0.00	0.00

Alcohols	2,3-Butanediol	12Al	0.00	0.00	0.00	20.29	14.83	20.83	0.00	0.00	0.00	0.00	0.00	0.00	15.04	10.72	15.83	0.00	0.00	0.00
Alcohols	2-Heptanol	13Al	16.96	17.16	24.30	12.14	6.88	9.77	0.00	0.00	0.00	10.84	14.00	17.88	0.00	4.71	6.35	0.00	0.00	0.00
Alcohols	2-Hexen-1-ol, (E)-	14Al	0.00	0.00	0.00	0.00	0.00	0.00	0.00	0.00	0.00	0.80	0.83	1.05	0.00	0.00	0.00	0.00	0.00	0.00
Alcohols	2-Propyl-1-pentanol	15Al	3.34	8.98	2.08	0.00	0.00	0.00	0.00	0.00	0.00	0.00	6.42	5.75	0.00	0.00	0.00	0.00	0.00	0.00
Alcohols	3-Hexen-1-ol	16Al	0.60	0.70	0.80	0.00	0.00	0.00	0.00	0.00	0.00	0.70	0.81	0.88	0.00	0.00	0.00	0.00	0.00	0.00
Alcohols	3-Octanol	17Al	1.51	1.57	1.62	0.00	0.00	0.00	0.00	0.00	0.00	0.37	0.77	1.02	0.00	0.00	0.00	0.00	0.00	0.00
Alcohols	5-Methyl-2-hexanol	1Al	17.39	17.88	25.37	0.00	6.99	10.11	0.00	0.00	0.00	11.32	14.29	18.34	0.00	4.71	6.65	0.00	0.00	0.00
Alcohols	6-Hepten-1-ol, 2-methyl-	18Al	0.37	0.24	0.25	0.00	0.00	0.00	0.00	0.00	0.00	0.28	0.17	0.15	0.00	0.00	0.00	0.00	0.00	0.00
Alcohols	Benzyl alcohol	19Al	2.70	2.70	2.22	3.81	4.18	3.00	0.04	0.03	0.03	1.60	1.59	1.33	1.65	1.23	0.86	0.00	0.00	0.00
Alcohols	Phenylethyl Alcohol	20Al	2.71	2.18	1.76	5.86	5.53	4.07	0.12	0.11	0.10	5.95	4.17	3.80	4.54	3.92	4.71	0.08	0.07	0.05
Aldehydes	2,4-Decadienal	1Ad	0.13	0.05	0.10	0.00	0.10	0.10	0.00	0.00	0.00	0.00	0.00	0.00	0.00	0.00	0.00	0.00	0.00	0.00
Aldehydes	2,4-Dodecadienal, (E,E)-	2Ad	0.19	0.10	0.12	0.00	0.00	0.00	0.00	0.00	0.00	0.00	0.00	0.00	0.00	0.00	0.00	0.00	0.00	0.00
Aldehydes	2,4-Heptadienal, (E,E)-	3Ad	1.19	1.30	0.00	0.00	1.19	0.46	0.00	0.00	0.00	0.00	0.00	0.00	0.00	0.00	0.00	0.00	0.00	0.00
Aldehydes	2,4-Nonadienal, (E,E)-	4Ad	0.21	0.10	0.12	0.10	0.11	0.09	0.00	0.00	0.00	0.10	0.08	0.11	0.31	0.71	0.46	0.00	0.00	0.00
Aldehydes	2,6-Nonadienal, (E,E)-	5Ad	0.38	0.20	0.40	0.21	0.20	0.22	0.00	0.00	0.00	0.35	0.14	0.38	0.72	0.62	0.51	0.00	0.00	0.00

Aldehydes	2-Furancarboxaldehyde, 5-methyl-	6Ad	0.00	0.00	0.00	0.00	0.00	0.00	8.01	7.82	7.46	0.00	0.00	0.00	0.00	0.00	0.00	8.29	7.91	8.26
Aldehydes	2-Nonenal, (E)-	7Ad	0.21	0.09	0.15	0.37	0.27	0.23	0.00	0.00	0.00	0.14	0.07	0.27	1.76	1.49	1.15	0.00	0.00	0.00
Aldehydes	2-Octenal, (E)-	8Ad	1.68	0.85	1.35	0.63	0.54	0.39	0.00	0.00	0.00	0.19	0.00	0.60	1.93	1.75	2.27	0.00	0.00	0.00
Aldehydes	2-Phenyl-2-butenal/ Benzeneacetaldehyde, .alpha.-ethylidene-	11Ad	0.00	0.00	0.00	0.07	0.05	0.06	0.00	0.00	0.00	0.00	0.00	0.00	0.00	0.00	0.00	0.00	0.00	0.00
Aldehydes	Benzaldehyde	9Ad	2.05	1.72	2.04	1.52	2.48	1.65	0.00	0.05	0.00	2.26	2.97	3.13	2.75	2.41	3.37	0.00	0.00	0.00
Aldehydes	Benzeneacetaldehyde	10Ad	4.55	7.61	4.01	0.00	0.00	0.00	0.00	0.00	0.00	17.09	15.27	11.02	0.00	0.00	0.00	0.00	0.00	0.00
Aldehydes	Furfural	12Ad	0.00	0.00	0.00	0.76	2.24	0.94	15.09	14.33	14.26	0.00	0.00	0.00	0.00	0.29	0.00	18.39	17.81	18.10
Aldehydes	Heptadecanal	13Ad	0.18	0.24	0.27	0.32	0.31	0.45	0.00	0.00	0.00	0.39	0.22	0.33	1.31	1.25	0.63	0.00	0.00	0.00
Aldehydes	Tetradecanal	14Ad	0.00	0.03	0.03	0.02	0.04	0.05	0.00	0.00	0.00	0.05	0.04	0.00	0.16	0.17	0.16	0.00	0.00	0.00
Alkanes	2-Furanmethanol, 5-ethenyltetrahydro-.alpha.,.alpha.,5-trimethyl-, cis-	1Ak	0.26	0.00	0.00	1.57	1.42	1.17	0.00	0.00	0.00	0.23	0.00	0.00	0.00	0.00	0.00	0.00	0.00	0.00
Alkanes	2-methyltetracosane	2Ak	0.00	0.04	0.00	0.00	0.00	0.00	0.00	0.00	0.00	0.00	0.00	0.00	0.19	0.17	0.17	0.00	0.00	0.00
Alkanes	Heneicosane	3Ak	0.00	0.02	0.02	0.00	0.00	0.07	0.14	0.02	0.01	0.00	0.00	0.00	0.00	0.00	0.00	0.02	0.00	0.00
Anhydrides	2,5-Furandione, 3,4-dimethyl-	1An	0.00	0.00	0.00	0.11	0.11	0.10	0.00	0.00	0.00	0.00	0.00	0.00	0.16	0.26	0.26	0.00	0.00	0.00

Esters	1,2-Benzenedicarboxylic acid, bis(2-methylpropyl) ester	1E	0.49	0.06	0.51	0.23	0.20	0.17	0.31	0.15	0.13	0.11	0.16	0.18	1.00	0.78	0.42	0.27	0.19	0.17
Esters	2-Butanone, 1-(acetyloxy)-	2E	0.00	0.00	0.00	0.00	0.00	0.00	1.24	1.27	1.15	0.00	0.00	0.00	0.00	0.00	0.00	1.05	1.06	1.09
Esters	2-Butenoic acid, 3-methyl-	3E	0.00	0.00	0.00	0.00	0.00	0.00	0.16	0.24	0.23	0.00	0.08	0.07	0.00	0.00	0.00	0.23	0.28	0.29
Esters	2-Ethylhexyl salicylate	4E	0.00	0.02	0.00	0.04	0.03	0.03	0.00	0.00	0.00	0.00	0.00	0.00	0.14	0.20	0.13	0.00	0.00	0.00
Esters	9,12,15-Octadecatrienoic acid, ethyl ester, (Z,Z,Z)-	5E	0.00	0.00	0.00	0.08	0.00	0.08	0.00	0.00	0.00	0.00	0.00	0.00	0.00	0.00	0.00	0.00	0.00	0.00
Esters	Acetic acid, 2-phenylethyl ester	6E	0.00	0.00	0.00	0.25	0.10	0.18	0.00	0.00	0.00	0.00	0.00	0.00	1.50	1.10	1.03	0.00	0.00	0.00
Esters	Acetic acid, phenylmethyl ester	7E	0.00	0.00	0.00	0.09	0.00	0.00	0.00	0.00	0.00	0.00	0.00	0.00	0.00	0.00	0.00	0.00	0.00	0.00
Esters	Benzeneacetic acid, ethyl ester	8E	0.45	0.32	0.19	0.16	0.13	0.00	0.00	0.00	0.00	0.21	0.31	0.29	0.32	0.42	0.00	0.00	0.00	0.00
Esters	Benzeneacetic acid, methyl ester	9E	0.24	0.12	0.08	0.00	0.00	0.00	0.00	0.00	0.00	0.21	0.15	0.11	0.00	0.00	0.00	0.00	0.00	0.00
Esters	Benzoic acid, ethyl ester	10E	0.16	0.10	0.10	0.16	0.20	0.12	0.00	0.00	0.00	0.00	0.00	0.00	0.00	0.00	0.00	0.00	0.00	0.00
Esters	Butanoic acid, 3-methyl-	11E	1.17	0.69	0.88	1.38	2.44	2.00	1.44	1.76	1.82	3.23	1.76	2.02	3.12	3.91	3.53	1.89	1.89	1.98
Esters	Hexadecanoic acid, ethyl ester	12E	0.08	0.09	0.10	1.01	0.71	1.38	0.04	0.04	0.04	0.07	0.00	0.06	0.37	0.35	1.22	0.06	0.03	0.00
Esters	Hexadecanoic acid, methyl ester	13E	0.22	0.20	0.23	0.18	0.18	0.20	0.00	0.00	0.00	0.18	0.08	0.11	0.16	0.12	0.17	0.00	0.00	0.00
Esters	Linalyl acetate	14E	3.86	0.00	0.00	0.00	0.00	0.00	0.00	0.00	0.00	0.07	3.32	2.75	0.00	0.00	0.00	0.00	0.00	0.00
Esters	Linoleic acid ethyl ester	15E	0.00	0.00	0.00	0.15	0.08	0.20	0.00	0.00	0.00	0.00	0.00	0.00	0.06	0.10	0.21	0.00	0.00	0.00

Esters	Propanoic acid, ethenyl ester	16E	0.00	0.00	0.00	0.00	0.00	0.00	0.58	1.20	1.11	0.00	0.00	0.00	0.00	0.00	0.00	0.86	1.13	1.07
Esters	Tetradecanoic acid, ethyl ester	17E	0.00	0.00	0.00	0.26	0.14	0.20	0.00	0.00	0.00	0.00	0.00	0.00	0.00	0.00	0.17	0.00	0.00	0.00
FFA	Ethyl 9-hexadecenoate	1FFA	0.00	0.00	0.00	0.00	0.00	0.16	0.00	0.00	0.00	0.00	0.00	0.00	0.00	0.00	0.00	0.00	0.00	0.00
FFA/Acids	Hexanoic acid	2FFA	0.00	0.11	0.13	1.75	1.59	1.32	0.00	0.00	0.00	0.00	0.00	0.14	3.06	4.76	2.09	0.00	0.00	0.00
FFA/Acids	Nonanoic acid	3FFA	1.06	0.60	0.78	1.25	2.28	1.81	0.00	0.00	0.00	2.98	1.61	1.79	2.78	3.43	3.10	0.00	0.00	0.00
FFA/Acids	Octanoic acid	4FFA	0.00	0.00	0.00	0.21	0.14	0.13	0.00	0.00	0.00	0.00	0.00	0.00	0.35	0.54	0.25	0.00	0.00	0.00
FFA/Acids	Pentanoic acid/Valeric acid	5FFA	0.00	0.00	0.00	0.29	0.38	0.26	0.00	0.00	0.00	0.00	0.00	0.00	0.49	0.80	0.62	0.00	0.00	0.00
FFA/Acids	Propanoic acid	6FFA	0.00	0.00	0.00	0.00	0.00	0.00	0.79	0.98	0.91	0.00	0.00	0.00	0.00	0.00	0.00	0.81	0.94	1.01
Furans	2,5-Dimethyl-4-hydroxy-3(2H)-furanone	1F	0.00	0.00	0.00	0.00	0.00	0.00	0.28	0.49	0.37	0.00	0.00	0.00	0.00	0.00	0.00	0.31	0.33	0.35
Furans	2-Furanmethanol	2F	0.00	0.00	0.00	0.00	0.00	0.00	31.02	28.74	24.95	0.08	0.00	0.00	0.00	0.00	0.00	28.76	26.55	26.40
Furans	2-Furanmethanol, 5-methyl-	3F	0.00	0.00	0.00	0.00	0.00	0.00	0.05	0.04	0.03	0.00	0.00	0.00	0.00	0.00	0.00	0.08	0.06	0.04
Furans	2-Furanmethanol, acetate	4F	0.00	0.00	0.00	0.00	0.00	0.00	1.42	1.18	1.22	0.00	0.00	0.00	0.00	0.00	0.00	1.00	0.95	1.09
Furans	3-Buten-2-one, 4-(2-furanyl)-	5F	0.00	0.00	0.00	0.00	0.00	0.00	0.03	0.04	0.04	0.00	0.00	0.00	0.00	0.00	0.00	0.04	0.04	0.04
Furans	4-Methyl-5H-furan-2-one	6F	0.00	0.00	0.00	0.00	0.00	0.00	0.05	0.06	0.05	0.00	0.00	0.00	0.00	0.00	0.00	0.04	0.04	0.04
Furans	5-Acetoxymethyl-2-furaldehyde	7F	0.00	0.00	0.00	0.00	0.00	0.00	0.00	0.29	0.28	0.00	0.00	0.00	0.00	0.00	0.00	0.00	0.24	0.25
Furans	5-Hydroxymethyldihydrofuran-2-one	8F	0.00	0.00	0.00	0.00	0.00	0.00	0.00	0.10	0.08	0.00	0.00	0.00	0.00	0.00	0.00	0.11	0.09	0.07

Furans	5-Hydroxymethylfurfural	9F	0.00	0.00	0.00	0.00	0.00	0.00	0.40	0.61	0.63	0.00	0.00	0.00	0.00	0.00	0.00	0.88	0.83	0.62
Furans	5-Methyl-furan-2-carboxylic acid (1H-[1,2,4]triazol-3-yl)-amide	10F	0.00	0.00	0.00	0.00	0.00	0.00	0.38	0.47	0.48	0.00	0.00	0.00	0.00	0.00	0.00	0.49	0.44	0.49
Furans	Benzofuran, 2,3-dihydro-	11F	0.00	0.00	0.00	0.00	0.00	0.00	0.15	0.21	0.24	0.00	0.00	0.00	0.00	0.00	0.00	0.45	0.28	0.23
Furans	Ethanone, 1-(2-furanyl)-	12F	0.00	0.00	0.00	0.00	0.00	0.00	2.11	2.17	2.07	0.00	0.00	0.00	0.00	0.00	0.00	2.36	2.28	2.34
Furans	Furan, 2,2'-[oxybis(methylene)]bis-	13F	0.00	0.00	0.00	0.00	0.00	0.00	0.08	0.12	0.11	0.00	0.00	0.00	0.00	0.00	0.00	0.11	0.09	0.12
Furans	Furan, 2-[(methyldithio)methyl]-	14F	0.00	0.00	0.00	0.00	0.00	0.00	0.11	0.08	0.00	0.00	0.00	0.00	0.00	0.00	0.00	0.00	0.00	0.00
Furans	Furfuryl formate	15F	0.00	0.00	0.00	0.00	0.00	0.00	0.67	0.58	0.55	0.00	0.00	0.00	0.00	0.00	0.00	0.55	0.49	0.59
Hydrocarbons	2-Hexene, 3,5,5-trimethyl-	1H	0.37	0.55	0.45	0.00	0.00	0.00	0.00	0.00	0.00	0.10	0.19	0.18	0.00	0.00	0.00	0.00	0.00	0.00
Hydrocarbons	Docosane	2H	0.06	0.06	0.08	0.08	0.04	0.04	0.00	0.03	0.00	0.05	0.00	0.07	0.13	0.10	0.00	0.00	0.00	0.00
Hydrocarbons	Heptadecane	3H	0.12	0.16	0.11	0.19	0.13	0.12	0.00	0.00	0.00	0.13	0.07	0.17	0.39	0.30	0.26	0.00	0.00	0.00
Hydrocarbons	Heptadecane, 2,6,10,15-tetramethyl-	4H	0.00	0.00	0.00	0.59	0.00	0.09	0.00	0.00	0.00	0.00	0.00	0.00	1.20	0.41	0.50	0.00	0.00	0.00
Hydrocarbons	Hexadecane, 2,6,10,14-tetramethyl-	5H	0.00	0.00	0.00	0.59	0.00	0.11	0.00	0.00	0.00	0.00	0.00	0.00	1.20	0.41	0.50	0.00	0.00	0.00
Hydrocarbons	Nonadecane	6H	0.10	0.09	0.10	0.00	0.00	0.00	0.05	0.00	0.00	0.12	0.10	0.00	0.00	0.00	0.00	0.00	0.00	0.00

Hydrocarbons	Tetradecane	7H	0.00	0.00	0.00	2.45	2.16	2.47	0.00	0.00	0.00	0.00	0.00	0.00	3.95	6.15	8.10	0.00	0.00	0.00
Ketones	1-(6-Methyl-2-pyrazinyl)-1-ethanone	1K	0.00	0.00	0.00	0.00	0.00	0.00	0.16	0.15	0.12	0.00	0.00	0.00	0.00	0.00	0.00	0.20	0.15	0.12
Ketones	1,2-Cyclopentanedione, 3-methyl-	2K	0.00	0.00	0.00	0.00	0.00	0.00	0.20	0.36	0.28	0.00	0.00	0.00	0.00	0.00	0.00	0.22	0.24	0.25
Ketones	2,5-Hexanedione	3K	0.00	0.00	0.00	0.00	0.00	0.00	0.15	0.23	0.19	0.00	0.00	0.00	0.00	0.00	0.00	0.21	0.13	0.19
Ketones	2-Butanone, 3,3-dimethyl-	4K	0.00	0.00	0.00	0.00	0.00	0.00	1.16	1.20	1.11	0.00	0.00	0.00	0.00	0.00	0.00	0.86	0.00	1.05
Ketones	2-Cyclopenten-1-one, 3-ethyl-2-hydroxy-	5K	0.00	0.00	0.00	0.00	0.00	0.00	0.05	0.10	0.08	0.00	0.00	0.00	0.00	0.00	0.00	0.07	0.07	0.07
Ketones	2-Pentadecanone, 6,10,14-trimethyl-	6K	0.07	0.09	0.03	0.34	0.15	0.19	0.00	0.01	0.01	0.10	0.03	0.04	2.49	1.42	0.93	0.03	0.02	0.02
Ketones	2-Propanone, 1-hydroxy-	7K	0.00	0.00	0.00	0.00	0.00	0.00	9.12	5.21	10.85	0.00	0.00	0.00	0.00	0.00	0.00	4.83	7.45	4.63
Ketones	5,9-Undecadien-2-one, 6,10-dimethyl-	8K	0.21	0.13	0.15	0.00	0.00	0.00	0.00	0.00	0.00	0.26	0.20	0.22	0.00	0.00	0.00	0.00	0.00	0.00
Ketones	Acetoin	9K	1.01	0.00	0.00	4.73	0.00	0.00	0.00	0.00	0.00	0.92	0.84	0.00	0.00	0.00	0.00	0.00	0.00	0.00
Lactones	2(3H)-Furanone, dihydro-5-pentyl-	1L	0.00	0.00	0.00	0.58	0.47	0.48	0.00	0.00	0.00	0.14	0.00	0.00	0.39	0.58	0.49	0.00	0.00	0.00
Lactones	2(5H)-Furanone	2L	0.00	0.00	0.00	0.00	0.00	0.00	0.88	0.86	0.71	0.00	0.00	0.00	0.00	0.00	0.00	0.69	0.70	0.70
Lactones	Butyrolactone	3L	0.00	0.00	0.00	1.62	1.31	1.31	2.38	1.97	1.84	0.00	0.00	0.00	0.00	0.00	0.00	1.09	1.13	1.17
Phenols	4-Hydroxy-2-methylacetophenone	1P	0.00	0.00	0.00	0.00	0.00	0.00	1.16	1.45	1.57	0.00	0.00	0.00	0.00	0.00	0.00	2.58	1.62	1.56

Phenols	Benzene, 2-methoxy-4-methyl-1-(1-methylethyl)-	2P	0.07	0.11	0.04	0.03	0.08	0.04	0.00	0.00	0.00	0.06	0.06	0.06	0.00	0.00	0.00	0.00	0.00	0.00
Phenols	Benzene, 4-ethenyl-1,2-dimethoxy-	3P	0.00	0.00	0.00	0.00	0.03	0.00	0.00	0.02	0.02	0.00	0.00	0.00	0.00	0.00	0.00	0.00	0.02	0.03
Phenols	Benzoic acid, 2-hydroxy-, ethyl ester	4P	0.16	0.00	0.00	1.48	1.47	1.24	0.00	0.00	0.00	0.48	0.00	0.00	0.37	0.00	0.00	0.00	0.00	0.00
Phenols	Ethanone, 1-(2-hydroxy-5-methylphenyl)-	5P	0.00	0.00	0.00	0.00	0.00	0.00	0.04	0.04	0.05	0.00	0.00	0.00	0.00	0.00	0.00	0.05	0.05	0.05
Phenols	Methyl salicylate	6P	3.93	2.42	3.95	15.02	16.33	10.69	0.00	0.00	0.00	9.03	1.46	0.84	5.29	4.06	2.81	0.00	0.00	0.00
Phenols	Phenol	7P	0.03	0.02	0.03	0.10	0.16	0.11	0.00	0.00	0.00	0.05	0.03	0.00	1.16	0.59	0.30	0.00	0.00	0.00
Phenols	Phenol, 3-methyl-	8P	0.00	0.00	0.00	0.00	0.00	0.00	0.04	0.06	0.06	0.00	0.00	0.00	0.00	0.00	0.00	0.05	0.06	0.06
Phenols	Phenol, 4-ethyl-2-methoxy-	9P	0.00	0.00	0.00	0.00	0.08	0.06	0.00	0.00	0.00	0.00	0.00	0.00	0.00	0.00	0.00	0.00	0.00	0.00
Pyrans	2H-Pyran-3-ol, 6-ethenyltetrahydro-2,2,6-trimethyl-	1Py	0.00	0.08	0.00	0.32	0.25	0.24	0.00	0.00	0.00	0.00	0.00	0.00	0.00	0.00	0.00	0.00	0.00	0.00
Pyrans	4H-Pyran-4-one, 2,3-dihydro-3,5-dihydroxy-6-methyl-	2Py	0.00	0.00	0.00	0.00	0.00	0.00	0.05	0.12	0.12	0.00	0.00	0.00	0.00	0.00	0.00	0.21	0.24	0.16
Pyrans	Maltol	3Py	0.00	0.00	0.00	0.00	0.00	0.00	0.95	1.13	0.85	0.00	0.00	0.00	0.00	0.00	0.00	1.09	1.18	1.54
Pyridines	3-Hydroxypyridine monoacetate	1Pi	0.00	0.00	0.00	0.00	0.00	0.00	0.41	0.50	0.49	0.00	0.00	0.00	0.00	0.00	0.00	0.51	0.52	0.50
Pyridines	3-Pyridinol	2Pi	0.00	0.00	0.00	0.00	0.00	0.00	0.16	0.21	0.21	0.00	0.00	0.00	0.00	0.00	0.00	0.26	0.23	0.24

Pyridines	4(H)-Pyridine, N-acetyl-	3Pi	0.00	0.00	0.00	0.00	0.00	0.00	0.51	0.50	0.44	0.00	0.00	0.00	0.00	0.00	0.00	0.52	0.46	0.43
Pyrroles	1H-Pyrrole, 1-(2-furanylmethyl)-	1Po	0.00	0.00	0.00	0.00	0.00	0.00	0.34	0.38	0.35	0.00	0.00	0.00	0.00	0.00	0.00	0.44	0.33	0.35
Pyrroles	1H-Pyrrole-2-carboxaldehyde	2Po	0.00	0.00	0.00	0.03	0.00	0.03	1.35	1.70	1.46	0.00	0.00	0.00	0.00	0.00	0.00	1.67	1.45	1.48
Pyrroles	1H-Pyrrole-2-carboxaldehyde, 1-methyl-	3Po	0.00	0.00	0.00	0.00	0.00	0.00	0.51	0.47	0.41	0.00	0.00	0.00	0.00	0.00	0.00	0.48	0.42	0.42
Pyrroles	2-Pyrrolidinone	4Po	0.00	0.00	0.00	0.07	0.07	0.09	0.08	0.06	0.07	0.00	0.00	0.00	0.00	0.00	0.00	0.03	0.00	0.00
Pyrroles	Ethanone, 1-(1H-pyrrol-2-yl)-	5Po	0.00	0.00	0.00	0.00	0.00	0.00	0.69	0.82	0.72	0.00	0.00	0.00	0.00	0.00	0.00	0.86	0.69	0.68
Pyrroles	Indole	6Po	0.00	0.00	0.00	0.00	0.00	0.00	0.15	0.17	0.21	0.00	0.00	0.00	0.00	0.00	0.00	0.34	0.23	0.20
Pyrroles	Pyrrole	7Po	0.00	0.00	0.00	0.00	0.00	0.00	1.16	0.63	0.60	0.00	0.00	0.00	0.00	0.00	0.00	1.09	0.62	0.64
Thiopenes	2-Thiophenecarboxaldehyde	1Th	0.00	0.00	0.00	0.00	0.00	0.00	0.12	0.00	0.10	0.00	0.00	0.00	0.00	0.00	0.00	0.12	0.11	0.11
Thiopenes	2-Thiophenemethanol	2Th	0.00	0.00	0.00	0.00	0.00	0.00	0.08	0.09	0.08	0.00	0.00	0.00	0.00	0.00	0.00	0.09	0.08	0.08

TF= End of fermentation

R= Roasted coffee samples
